# Promoting Fruit and Vegetable Consumption Among Members of Black Churches, Michigan and North Carolina, 2008–2010

**DOI:** 10.5888/pcd10.120161

**Published:** 2013-03-14

**Authors:** Marlyn Allicock, La-Shell Johnson, Lucia Leone, Carol Carr, Joan Walsh, Andi Ni, Ken Resnicow, Michael Pignone, Marci Campbell

**Affiliations:** Author Affiliations: La-Shell Johnson, Lucia Leone, Carol Carr, Joan Walsh, Andi Ni, Michael Pignone, Marci Campbell, University of North Carolina at Chapel Hill, Chapel Hill, North Carolina; Ken Resnicow, University of Michigan, Ann Arbor, Michigan.

## Abstract

**Introduction:**

Evidence-based health promotion programs that are disseminated in community settings can improve population health. However, little is known about how effective such programs are when they are implemented in communities. We examined community implementation of an evidence-based program, Body and Soul, to promote consumption of fruits and vegetables.

**Methods:**

We randomly assigned 19 churches to 1 of 2 arms, a colon cancer screening intervention or Body and Soul. We conducted our study from 2008 through 2010. We used the RE-AIM (reach, effectiveness, adoption, implementation, and maintenance) framework to evaluate the program and collected data via participant surveys, on-site observations, and interviews with church coordinators and pastors.

**Results:**

Members of 8 churches in Michigan and North Carolina participated in the Body and Soul program. Mean fruit and vegetable consumption increased from baseline (3.9 servings/d) to follow-up (+0.35, *P* = .04). The program reached 41.4% of the eligible congregation. Six of the 8 churches partially or fully completed at least 3 of the 4 program components. Six churches expressed intention to maintain the program. Church coordinators reported limited time and help to plan and implement activities, competing church events, and lack of motivation among congregation members as barriers to implementation.

**Conclusions:**

The RE-AIM framework provided an effective approach to evaluating the dissemination of an evidence-based program to promote health. Stronger emphasis should be placed on providing technical assistance as a way to improve other community-based translational efforts.

## Introduction

Adequate consumption of fruits and vegetables, 5 or more servings per day, may reduce the risk of several chronic diseases (1,2) and prevent approximately 30% of cancer deaths (3). However, many Americans are not meeting the dietary recommendations of the US Department of Agriculture (1,4,5), and blacks appear to have less healthful diets than whites (5,6). Data for 2009 indicate only 22.4% of blacks reported eating recommended amounts of fruits and vegetables (7). Therefore, effective behavioral interventions directed at increasing fruit and vegetable consumption are needed.

In general, behavioral interventions that perform well in research settings rarely reach the general public (8). Once an intervention is disseminated for implementation by community members, little information becomes available about the process of implementation (ie, how the intervention is used and modified in real-world settings). This knowledge gap has raised concern at the national level (9). The National Cancer Institute’s (NCI’s) strategic plan highlights the importance of enhancing delivery and use of evidence-based interventions (9), and the Centers for Disease Control and Prevention affirms that the future health of the nation will be determined by how effectively health disparities are reduced and eliminated in communities (10).

Several reviews support the use of church-based health promotion programs (11,12), especially those directed at minority populations (12–15). We developed a program, Body and Soul, for improving dietary behaviors by black church members (16,17). This program is disseminated nationally by NCI and is cited as 1 of its Research-Tested Intervention Programs (http://rtips.cancer.gov/rtips/index.do). However, we know little about its implementation fidelity. The RE-AIM (reach, effectiveness, adoption, implementation, and maintenance) framework (18) assesses interventions that are developed, implemented, and evaluated in research-controlled environments and then disseminated for widespread use in community settings (18–22), including churches (23). We used RE-AIM (18) to assess implementation of Body and Soul in churches in 2 states, Michigan and North Carolina, as part of the larger Body and Soul randomized, controlled trial.

## Methods

### Design

The Action in Churches in Time to Save Lives (ACTS) of Wellness program was a randomized, controlled trial conducted in black churches in North Carolina and Michigan from 2008 through 2010 to increase colorectal cancer screening and physical activity. Participants at 10 intervention churches received 2 newsletters about physical activity and colorectal cancer screening tailored to their individual behavioral characteristics and up to 3 motivational interviewing counseling calls with peer counselors. Churches in the control arm (n = 8) implemented Body and Soul. Technical assistance from research staff to each church was limited to a 2-hour meeting discussing program components, resources needed, and logistics. Thus, this project provided an opportunity to evaluate the implementation of Body and Soul under real-world conditions. The institutional review board of the University of North Carolina at Chapel Hill approved this study.

### Body and Soul program

A detailed description of Body and Soul was published previously (16,17). Early studies found it was effective at increasing fruit and vegetable consumption among church members (16,24–26), and NCI adopted it in 2003 for national dissemination to black churches. We conducted a subsequent randomized, delayed-design study in 16 churches nationwide in 2006 to assess the efficacy of Body and Soul as churches adopted the program on their own (27); that study found no significant effect on fruit and vegetable consumption and raised questions regarding program components, program dosage delivered, and barriers and facilitators to implementation. The study described in this article sought to address these questions.

The Body and Soul program has 4 “pillars”: pastoral involvement (support the church’s health-related efforts), educational activities (eg, offer opportunities to sample and prepare fruits and vegetables), church environmental changes (eg, set up policies about serving healthful foods at church events), and peer counseling (provide one-on-one support from trained volunteer church members by using motivational interview principles [28] with training provided via a DVD and detailed implementation manuals).

### Settings and participants

#### Church recruitment and eligibility

We conducted a rolling recruitment and randomization of churches in urban areas in Flint, Michigan, and in Wake, Durham, and Guilford counties in North Carolina from January 2008 through August 2009. In North Carolina, recruitment was via mail, telephone calls, and meetings with church pastors and interested members. In Michigan, churches were recruited through their affiliation with Faith Access to Community Economic Development (FACED), a community partner working in black communities.

To be eligible to participate, churches were required to have a predominantly black congregation, 250 or more active adult members, and 100 or more members aged 50 years or older. They also had to agree to randomization. We contacted 40 churches in Michigan and North Carolina (Figure). Of these, 14 declined or did not respond and 26 expressed interest; of the 26 that expressed interest, 7 did not meet eligibility criteria. Nineteen churches enrolled: 12 Baptist, 3 African Methodist Episcopalian (AME), 1 AME Zion, 1 Catholic, and 2 nondenominational churches. We randomized 10 churches to a colon cancer screening intervention and 9 to Body and Soul. One of the 9 Body and Soul churches withdrew from the study after baseline survey completion because of organizational changes, and we excluded its data from analyses. Pastors from each participating church signed a contract listing the responsibilities of the church and research team. Each pastor then assigned a church coordinator to manage program logistics. We offered participating churches the following incentives: $300 at sign-on, $300 (plus $150 for the church coordinator) at completion of baseline, and $300 (plus $150 for the coordinator) at completion of follow-up plus $200 for follow-up rates of 90% or higher.

**Figure:**
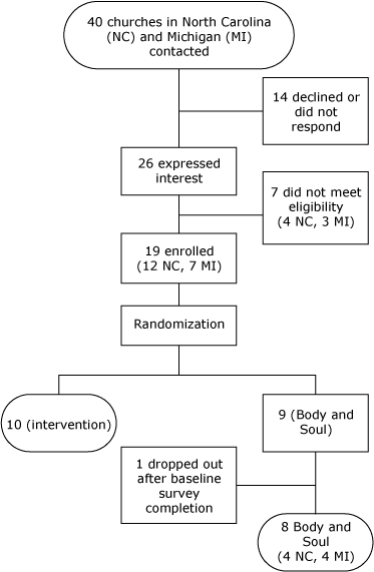
Process of recruitment of churches in North Carolina and Michigan to participate in a program to promote fruit and vegetable consumption among members of black churches in Michigan and North Carolina, 2008–2010.

#### Participant recruitment and eligibility

Church coordinators arranged meetings with potential participants, usually after Sunday services. Study staff then presented information about the study, and members willing to participate provided consent and completed the baseline survey. Church members were eligible if they spoke English and were aged 50 years or older (the second criterion was due to the primary aim of ACTS, colon cancer screening). We offered incentives for participants at baseline, including meals provided at survey collection, pedometers for intervention participants, and kitchen aprons for control participants. At follow-up, intervention participants received aprons and control participants received pedometers. Although we tried to collect all follow-up surveys 6 months after baseline, some data collection occurred as late as 12 months post-baseline because of scheduling conflicts at the churches.

### Program evaluation and measures

Using both quantitative and qualitative data sources, we examined the 5 core components of RE-AIM to understand implementation. *Reach* is the estimated proportion of participating eligible church members. Data were available from 6 of the 8 churches about the number of eligible congregants on which to base this calculation (some membership data were missing because the churches’ bookkeeping was out of date). We determined *effectiveness*, the positive effect of the program on fruit and vegetable consumption, by using 2 measures included on baseline and follow-up surveys: a 9-item questionnaire about fruit and vegetable consumption adapted from a 35-item measure for US Southern blacks (29) that measured frequency of intake during the previous month, and a 2-item measure (How many servings of fruits do you eat each day?) and a similar question for vegetables (29) that assessed usual fruit and vegetable intake. *Adoption* is the proportion and representativeness of settings and of the people who implement the program (a report of adoption is not included because assignment to Body and Soul was random). *Implementation* is the extent to which the intervention was delivered as intended (assessed through follow-up interviews with coordinators or pastors, observations of peer counselor training, and process questions on the follow-up participant survey). *Maintenance* is the long-term adoption and implementation of the intervention (evaluated as plans for continuing Body and Soul assessed in follow-up telephone interviews with pastors or coordinators).

### Statistical analysis

We used SAS 9.2 (SAS Institute, Inc, Cary, North Carolina) to analyze data. We generated descriptive statistics for all variables and summarized continuous variables by mean and standard deviation and categorical variables by frequency and percentage. Because the randomization was based on churches, we used linear mixed-effect models with random intercept to control for the clustering effect of church. A 2-sided *P* value of ≤.05 was considered significant.

Trained research staff conducted interviews, which lasted approximately 30 minutes. Staff audio recorded and transcribed telephone interviews with coordinators (n = 6) and pastors (n = 5). Pastor interviews asked about awareness of Body and Soul activities and pastors’ involvement in implementation. Coordinators were asked about perceived facilitators and barriers to implementation, activities conducted, and changes to organizational policies and procedures as a result of implementing Bodyand Soul. Two team members conducted thematic analyses to identify recurring themes. They compared codes, resolved differences through discussion with project team members, and summarized key findings in relation to program implementation and maintenance.

## Results

### Reach

Of the 8 Body and Soul churches, 4 were in North Carolina and 4 in Michigan. Data for the number of active congregants aged 50 years or older were available for 6 churches and totaled 730 (min 55, max 200), and 302 congregants from these 6 churches (min 32, max 44) completed the baseline and follow-up surveys. Overall reach (proportion of eligible congregants who participated in the full study) is therefore estimated at 32% (231/730), 23% of the women and 9% of the men congregants. Study participants were mainly women (71%), married or partnered (51.5%), had some college education or a college degree (71%), and had annual household incomes of less than $50,000.

### Effectiveness

Baseline mean fruit and vegetable consumption for Body and Soul participants (n = 302) was 4.07 servings per day using the 9-item scale and 3.89 servings per day using the 2-item scale. Servings per day at follow-up was higher when the 9-item measure was used (mean, +0.14 servings; standard deviation [SD], 3.79; *P* = .63), but this increase was not significant. However, with the 2-item measure, the increase was small but significant (mean, +0.35 servings; standard deviation [SD], 1.97; *P* = .04).

### Implementation

Through pastor and coordinator interviews, all churches reported pastoral support; 4 churches made environmental changes, and 6 churches conducted at least 1 or 2 educational activities (2 reported 3–5 activities). The research team observed peer counselor training at 7 churches; only 2 churches initiated the peer counseling program. Two churches implemented 2 of the 4 program pillars, 3 implemented 3 pillars, and 3 implemented all pillars. The overall intervention period varied from 9 to 17 months depending on church personnel changes, the coordinator’s availability, and other events in the church taking priority over study activities.

**Implementation of pillar 1 (pastoral support).** All 8 churches had pastoral support via pastoral consent for the program and pastor’s attendance at events; some pastors delivered health-centered messages during sermons. One pastor stated, “The body and soul are intertwined, and it would be pathetic of me to just talk about someone's soul and not touch on their body.” Pastoral counseling to individual members about health (n = 8) and encouraging church members from the pulpit about weight loss (n = 3) were other indicators of pastoral support.

**Implementation of pillar 2 (educational activities).** Six of the 8 churches reported conducting church-wide educational activities including food tastings, free food fairs that showcased healthful food options and preparation, substituting more healthful foods at church events (eg, Sunday breakfasts), and placing inserts about healthful eating in church bulletins. Church coordinators also provided sample menus, recipes, and videos to congregants. In addition to these nutrition activities, 3 churches began fitness programs emphasizing weight loss (eg, biggest loser, walking club). At follow-up, 60% of participants reported attending or being exposed to health education activities. Of those, 76% indicated that the events focused on fruits and vegetables. After controlling for baseline demographics, fruit and vegetable consumption was higher at follow-up (*P* = .001) among participants who recalled Body and Soul events (4.56 servings/d vs 3.72 servings/d, 2-item scale; 4.33 vs 4.06, 9-item scale) (Table).

**Table:** Relationship Between Exposure to, and Perceptions About, Body and Soul Program in Black Churches (N = 302) and Primary and Secondary Outcomes at 6-Month Follow-up, Michigan and North Carolina, 2008–2010

Process Variable	% (n)	Average Servings of Fruits and Vegetables Per Day, 2-Item Scale (SD)	Average Servings of Fruits and Vegetables Per Day, 9-Item Scale (SD)
**Recall of Body and Soul church events**			
Yes	60.1 (179)	4.56 (2.18)	4.33 (2.84)
No	39.9 (119)	3.72 (1.96)	4.06 (3.80)
*P* value^a^	NA	.001	.60
**If yes, number of events attended**			
0	15.8 (28)	5.05 (2.34)	5.03 (3.57)
1	33.3 (59)	3.62 (1.80)	3.65 (2.85)
2	24.9 (44)	4.84 (2.28)	4.37 (2.39)
≥3	26.0 (46)	5.16 (2.12)	4.67 (2.71)
*P* value^a^		.07	.10
**Recall talking with peer counselor^b^ (telephone/in person)**			
Yes	12.9 (39)	4.45 (2.23)	4.26 (2.37)
No	87.1 (263)	4.19 (2.11)	4.21 (3.35)
*P* value^a^	NA	.76	.70
**If yes, number of calls/conversations**			
1 to 2	63.6 (21)	4.14 (2.32)	4.49 (2.59)
3 to 4	24.2 (8)	5.38 (2.66)	4.51 (2.71)
>4	12.1 (4)^c^	4.13 (1.03)	3.81 (1.45)
*P* value^a^	NA	.70	NA^b^

Abbreviations: SD, standard deviation; NA, not applicable.

a Controlling for baseline intake. *P* values are from Wald tests in linear mixed effect models.

b Too few subjects to calculate *P* value.

c Numbers do not total 100 because of rounding.

**Implementation of pillar 3 (environmental change).** Four churches reported making environmental changes, such as serving more healthful foods, substituting fruit for doughnuts at church breakfasts, serving water instead of sugary drinks, and using fruit in gift baskets.

**Implementation of pillar 4 (peer counseling).** Seven of the 8 churches trained volunteers to serve as peer counselors, but only 2 churches had begun to roll out the program to congregation members. We trained 26 volunteer peer counselors (mean age, 57 years; SD, 9.9); 22 were women. Average number of peer counselors per church was 4 (range, 2–5). Research staff observed 5 trainings that church coordinators conducted. Observers found that the trainings lasted approximately 2 hours (range 2–2.5 hours) and trainers used 2 of the 4 DVD modules we provided. Coordinators were able to use the DVDs and manuals with ease. In debriefing sessions, peer counselor trainees rated the DVD and training materials favorably. Only 32% of participants across all the churches reported being aware of peer counseling, and of these, 13% talked with a peer counselor. We found no relationship between fruit and vegetable consumption and report of talking to a peer counselor, for either fruit or vegetable measure.

### Maintenance

At follow-up, 6 coordinators reported intentions to continue the program, but 1 stipulated that extra staff would be needed to do so. The 2 churches that had no plans for continuing cited low interest of congregants, competing church programs, and lack of personnel. Five of the 8 pastors were pleased with the program and wanted to see it continue. Maintaining healthful food alternatives during church events and continuing to provide information about healthy living were actions coordinators believed they could sustain without additional cost or effort.

### Implementation challenges

Challenges reported by pastors and coordinators included lack of time to plan and implement activities, difficulty scheduling events at convenient times, competing church programs, inability to reach congregants consistently, and congregant lack of motivation. Four churches made no environmental or policy changes because coordinators in these churches were focused on implementing other aspects of the study (recruiting peer counselors, activity planning). Another challenge coordinators noted was that some participants did not want to disclose personal information to peer counselors. Some coordinators said they lacked confidence to implement and promote the program, leading to delays in start-up and failure to delegate tasks. Implementing the program without ongoing technical assistance was cited as a barrier (technical support was provided only at program onset).

## Discussion

This study focused on understanding the implementation of an evidence-based program in response to the knowledge gap about the uptake of programs disseminated to communities. We used RE-AIM to provide a comprehensive assessment of implementation of Body and Soul. Program reach was low and may have been hindered by the parent study’s parameters, which required that participants be 50 years or older given the focus on colorectal cancer screening. Although data for younger participants were not collected, 3 of the 4 Body and Soul components were adopted on a church-wide basis (peer counseling excluded) and not limited to those 50 years or older. It is possible that other members benefited and participated in these study components, but data on their participation were not captured. The program showed overall improvements in fruit and vegetable consumption (2-item measure) among congregation members. Regarding implementation, all churches had pastoral involvement, all conducted at least 1 church-wide nutrition event, and half achieved an environmental change. Only 2 churches began peer counseling. Given the low intensity of the intervention as it was delivered, the small increase in fruit and vegetable consumption is substantial. Regarding maintenance, all but 2 churches indicated their intention to continue some aspect of the program.

Our findings of low reach and suboptimal implementation replicate those of a previous evaluation assessing independent church implementation of health promotion programs (27). It is evident that churches need additional support, such as continued technical assistance and training for church coordinators, to help with implementation and to ensure program institutionalization. We believe that additional or different strategies are needed to encourage implementation. Despite enthusiasm about the need for health promotion programs, coordinators cited need for help with program planning and technical assistance to support program activities. Program planning and implementation relied on volunteers. Coordinators were given a small monetary incentive for their time; however, Body and Soul is intended to be volunteer-based. Coordinators also reported that congregants seemed to lack interest in participating. Adoption of the program may need to be a choice made across the congregation instead of by the pastor alone, as was the case in this study.

Despite these barriers, we were interested to see that 3 churches expanded the program by adding a physical activity component. Promoting fruit and vegetable consumption may have had the unintentional effect of encouraging physical activity programs. Fitness activities may have taken time and resources from the Body and Soul nutrition program but may also have encouraged participation given congregants’ interest in weight management.

This study had several limitations. The geographic dispersion of churches limited our ability to conduct direct observation of some program activities. A second limitation may have been in the measures used to evaluate fruit and vegetable consumption. The 9-item measure included specific foods that were culturally appropriate and was validated in a black church-based population (29). The 2-item measure captured total intake for fruits and vegetables. Perhaps it was easier for participants to estimate their total intake rather than provide specifics regarding individual food items. It is also likely that recall bias may have been a factor. Finally, the 6-month time frame we originally designated was not adequate for churches to implement all program components. Because of the people and time resources available and competing church activities, the actual time churches needed to complete each step, from obtaining baseline surveys to final survey completion, was a minimum of 12 to 17 months.

Strong evidence from studies continues to accumulate for the efficacy of promoting health behavior changes through black churches (19,23,24,26,30). Because none of these studies were in the dissemination phase, published literature provides little guidance about how best to evaluate and promote implementation when communities adopt these programs on their own. The Health-e-AME (23), an intervention to promote physical activity in churches via trained volunteer health directors, used the RE-AIM model to understand program potential for translation beyond the tightly controlled research setting. Findings showed that the intervention was moderately implemented and had no significant effect on physical activity; however, use of the RE-AIM design afforded these researchers a model for conducting health promotion with dissemination in mind to allow for understanding and addressing barriers to program implementation.

The RE-AIM model was useful in understanding impact and implementation and for gaining insight about maintenance — factors relevant to external validity. This program was designed for black congregations, but it is adaptable to other racial, ethnic, or religious populations. With any faith-based community, understanding the context in which a program operates is a factor for success of health promotion efforts. We included a diverse sample of denominations and selected churches in 2 geographic regions to improve generalizability of findings. Future studies should allow longer intervention periods; find innovative ways to build interest across the population, such as including other behaviors of interest; and provide technical assistance on implementing all program components (eg, partnerships with successful implementers, expanded training materials that address the gaps identified). Research that addresses these factors could provide a true evaluation of effectiveness of the disseminated program.
